# Discovery of genistein derivatives as potential SARS-CoV-2 main protease inhibitors by virtual screening, molecular dynamics simulations and ADMET analysis

**DOI:** 10.3389/fphar.2022.961154

**Published:** 2022-08-25

**Authors:** Jiawei Liu, Ling Zhang, Jian Gao, Baochen Zhang, Xiaoli Liu, Ninghui Yang, Xiaotong Liu, Xifu Liu, Yu Cheng

**Affiliations:** ^1^ Center for Drug Innovation and Discovery, College of Life Science, Hebei Normal University, Shijiazhuang, China; ^2^ School of Chemical Technology, Shijiazhuang University, Shijiazhuang, China; ^3^ College of Plant Protection, Southwest University, Chongqing, China

**Keywords:** SARS-CoV-2, genistein, molecular docking, molecular dynamics simulation, ADMET

## Abstract

**Background:** Due to the constant mutation of virus and the lack of specific therapeutic drugs, the coronavirus disease 2019 (COVID-19) pandemic caused by the severe acute respiratory syndrome coronavirus 2 (SARS-CoV-2) still poses a huge threat to the health of people, especially those with underlying diseases. Therefore, drug discovery against the SARS-CoV-2 remains of great significance.

**Methods:** With the main protease of virus as the inhibitor target, 9,614 genistein derivatives were virtually screened by LeDock and AutoDock Vina, and the top 20 compounds with highest normalized scores were obtained. Molecular dynamics simulations were carried out for studying interactions between these 20 compounds and the target protein. The drug-like properties, activity, and ADMET of these compounds were also evaluated by DruLiTo software or online server.

**Results:** Twenty compounds, including compound **11**, were screened by normalized molecular docking, which could bind to the target through multiple non-bonding interactions. Molecular dynamics simulation results showed that compounds **2**, **4**, **5**, **11**, **13**, **14**, **17**, and **18** had the best binding force with the target protein of SARS-CoV-2, and the absolute values of binding free energies all exceeded 50 kJ/mol. The drug-likeness properties indicated that a variety of compounds including compound **11** were worthy of further study. The results of bioactivity score prediction found that compounds **11** and **12** had high inhibitory activities against protease, which indicated that these two compounds had the potential to be further developed as COVID-19 inhibitors. Finally, compound **11** showed excellent predictive ADMET properties including high absorption and low toxicity.

**Conclusion:** These in silico work results show that the preferred compound **11** (**ZINC000111282222**), which exhibited strong binding to SARS-CoV-2 main protease, acceptable drug-like properties, protease inhibitory activity and ADMET properties, has great promise for further research as a potential therapeutic agent against COVID-19.

## Introduction

In recent years, the coronavirus disease 2019 (COVID-19) has had a severe negative impact on the world’s health and economy, which is caused by severe acute respiratory syndrome coronavirus 2 (SARS-CoV-2) (Hu et al., 2021). At present, the main measures to deal with the disease are vaccination and symptomatic treatment with drugs, but the existing drugs have limited inhibitory effect on the virus (Murugesan et al., 2021; Male 2022). Moreover, as the virus continues to mutate, its infectivity is enhanced and the effectiveness of vaccines can be compromised, and the COVID-19 pandemic still poses a huge threat to the health of people, especially those with underlying diseases (Marjot et al., 2021; Araf et al., 2022). Therefore, the rapid discovery and development of novel, effective and safe drugs for the treatment of COVID-19 remains current the focus of research in countries around the world.

**FIGURE 1:**
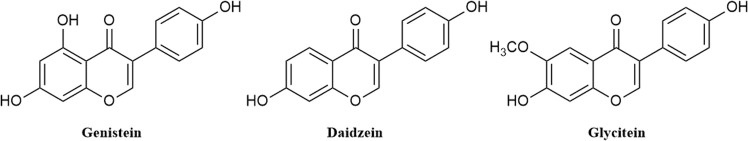
Structure of daidzein, genistein, and glycitein.

Soy isoflavones isolated from soybeans have received extensive attention for their ability to prevent osteoporosis (Rodríguez et al., 2022), inhibit the growth of cancer cells (Yamashita et al., 2022), reduce the risk of cardiovascular disease (Im and Park, 2021) and relieve menopausal symptoms (Chen and Chen, 2021). They are a class of flavonoids with C6-C3-C6 as the nucleus and researchers from various countries have isolated 12 types of soy isoflavones from plants. These soy isoflavones can be divided into four categories, which are free aglycones (genistein, daidzein, and glycitein) (Figure 1), β-glycosides (genistin, daidzin, and glycitin), acetyl β-glycosides (acetyl genistin, acetyl daidzin, and acetyl glycitin) and malonyl β-glycosides (malonyl genistin, malonyl daidzin, and malonyl glycitin). (Kim et al., 2022) Naturally, 50%–90% of soy isoflavones in soybeans exist in the form of glycosides, but studies have shown that daidzein, genistein and glycitein are the main active substances for soybean isoflavones to exert their pharmacological functions, among which genistein has the highest activity. (Izumi et al., 2000; Handa et al., 2014) Genistein can block the proliferation of cancer cells by various mechanisms, such as upregulating p21 level (Park et al., 2019) and inhibiting the activity of tyrosine-specific kinase (Uckun et al., 1995). It also exhibits anti-angiogenic and antioxidant activities and is used to prevent heart disease and cardiovascular disease. (Jaiswal et al., 2019; Nazari-Khanamiri and Ghasemnejad-Berenji, 2021) In addition, the viral RNA transcripts and protein synthesis such as rotavirus can also be inhibited by genistein (Huang et al., 2015). Therefore, genistein has potential as a lead compound for screening of antiviral drug molecules.

Drug discovery is a capital- and time-intensive process, and an efficient way to achieve this goal is through computer-aided drug design in the preclinical phase of drug discovery (Gurung et al., 2021; Salman et al., 2021). Among the many methods, virtual screening is the main method of computer-aided drug design, among which, molecular docking methods have been widely used in virtual screening to help simplify the search, especially when the three-dimensional crystal structure of the protein receptor is available (Khan et al., 2018; Gentile et al., 2022). The purpose of molecular docking is to predict the binding conformation and intermolecular affinity of ligands and receptors. However, the use of single-molecule docking methods is prone to false positive results, that is, the sampling algorithm cannot generate the correct binding conformation or the scoring function cannot pick out the correct binding conformation after scoring and sorting (Pagadala et al., 2017; Vidal-Limon et al., 2022). The use of different molecular docking software has the potential to enhance the correct rate of molecular docking results and some studies have found that among more than 10 molecular docking software used so far, LeDock has the best sampling performance, while AutoDock Vina has the best scoring performance (Wang et al., 2016; Abelyan et al., 2020; Santos et al., 2020). Therefore, the simultaneous use of LeDock and AutoDock Vina is beneficial to improve the accuracy of molecular docking results.

Furthermore, it is well known that most molecular docking software estimates binding energies through force field calculations guided by quantum mechanics and experimental data. However, precise binding energies can only be determined by *ab initio* methods such as DFT and molecular dynamics simulations (Honarparvar et al., 2014; Sabe et al., 2021). Because aspects such as protonation and solvation are taken into account, molecular dynamics simulations can yield more information about these preliminary results. (Sabe et al., 2021) Molecular dynamics simulation is one of the necessary follow-up computational methods for complete drug virtual screening technology, and it is a supplement to molecular docking. More importantly, in addition to binding energies, molecular dynamics simulation can also validate the protein-ligand complex results obtained by molecular docking by analyzing the stability of the established complex, the interactions between atoms, and the volatility of the simulation system (Menchon et al., 2018). Therefore, the combined use of molecular docking and molecular dynamics simulations contributes to the speed and accuracy of drug discovery. At present, many biologically active molecules, including anti-coronavirus drugs, have been discovered in this way. (Kumar et al., 2020) In addition to the affinity between the drug molecule and the target, some drug-related properties of the molecular entity itself should also be considered, such as drug-likeness, absorption, distribution, metabolism, excretion, and toxicity.

**FIGURE 2:**
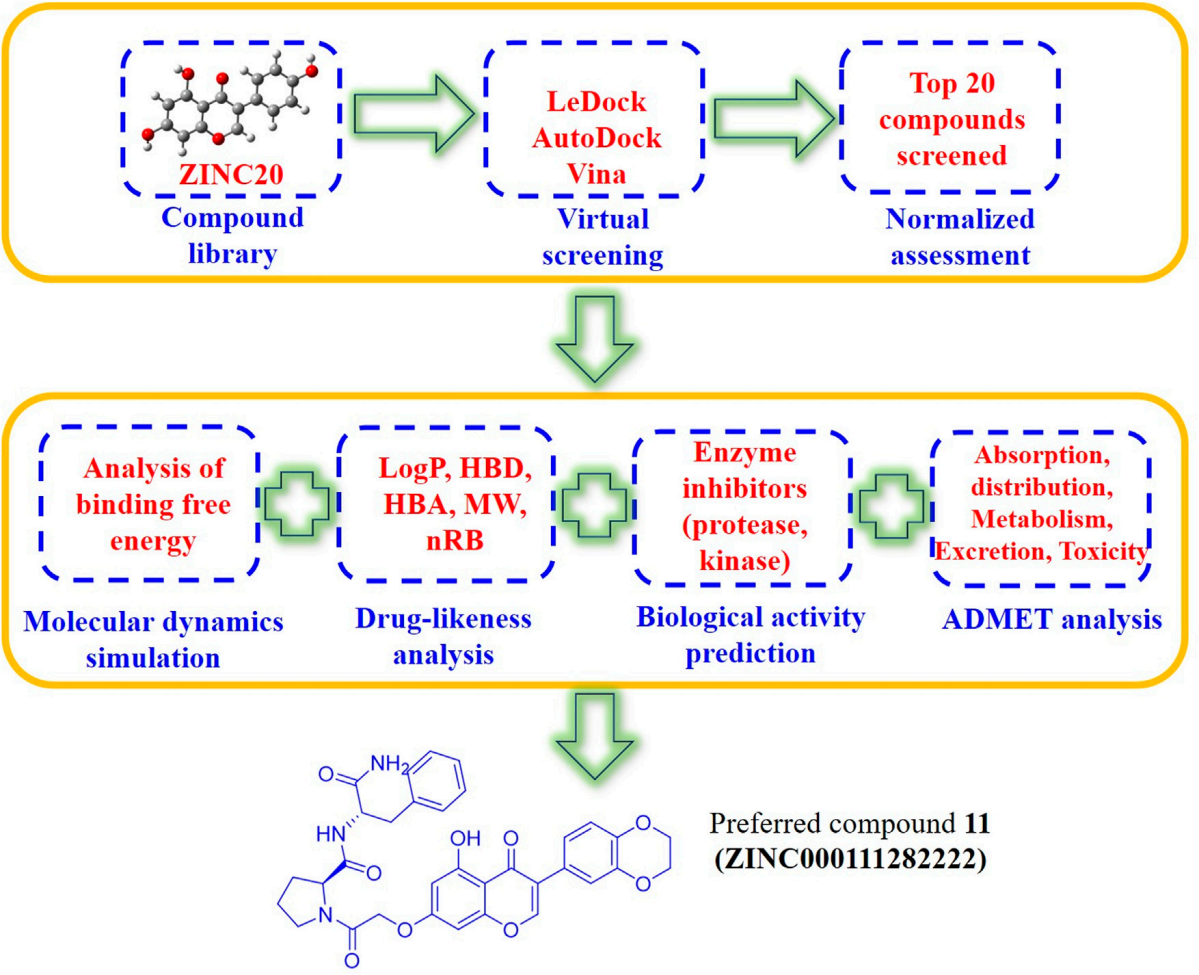
Schematic diagram of the discovery of genistein-based anti-SARS-CoV-2 drug molecules.

At present, the COVID-19 pandemic still poses major challenges to the public health of countries around the world. Scientists from all over the world have made great efforts and achieved remarkable results in the research on the structural biology, epidemiology and antiviral intervention of SARS-CoV-2. But there are still few drugs that are effective against viral infections. Based on the above considerations, genistein have a wide range of biological activities, molecular docking can quickly realize virtual screening, and molecular dynamics simulation as an effective supplement to molecular docking can reveal the noncovalent interactions between drug molecules and target proteins at the molecular level. In this manuscript, genistein, the main biologically active ingredient in isoflavones, was used as the parent structure, and genistein-derived compounds were enriched from ZINC20 library. The enriched isoflavone compounds were molecularly docked with the main protease of SARS-CoV-2 by two different molecular docking methods (LeDock and AutoDock Vina). Finally, further molecular dynamics simulations were performed on the top 20 compounds screened by molecular docking, and their activity, absorption, distribution, metabolism, excretion, and toxicity were also calculated. (Figure 2) The above research results will provide theoretical guidance for the development of novel anti-COVID-19 drugs based on genistein and insights into the mechanism of drug action.

## Computational method

### Receptor preparation

Recent studies have shown that main protease (M^pro^) is the most promising drug target against SARS-CoV-2. (Zhang et al., 2020) In this manuscript, the three-dimensional crystal structure (PDB ID: 6LU7) of the main protease of SARS-CoV-2 in complex with a peptide-like inhibitor **N3** was obtained from the RCSB PDB (https://www.rcsb.org/). (Hatada et al., 2020) The crystallographic structure was imported into Discovery Studio 2019 Client program to detect the centre of the active site according to the position of **N3** in the structure [center of the active site: x = −10.73, y = 12.42, and z = 68.82 (Å)]. AutoDockTools-1.5.6 was used to prepare the protein by removing water and **N3** from the active site, adding polar hydrogen atoms, and converting the protein PDB files to a PDBQT format.

### Ligand preparation

ZINC20 is a small molecule database commonly used for drug virtual screening, with more than 1.4 billion compound molecules. Arthor (https://arthor.docking.org) is a small molecule structure search tool newly added to ZINC20 and is by far the fastest method for substructure and pattern searches at the atomic level. (Irwin et al., 2020) In this manuscript, the nucleus of genistein was used as the initial structure, and the approximate derivative structure was searched in Arthor, and a total of 9,650 derivatives of genistein were obtained. Since 36 derivatives of genistein contained atoms that were not recognized by the docking software, only 9,614 derivatives of genistein in PDB format were used as input files for subsequent molecular docking of LeDock and AutoDock Vina.

### Molecular docking

For AutoDock Vina, the grid box’s center points and dimensions were set to target the active site of the main protease, with the center at x = −10.73, y = 12.42, and z = 68.82 (Å), and the grid box’s dimensions set to X: 30, Y: 30, and Z: 30 (Å). The parameters of exhaustiveness and num_modes were considered to be 16 and 20, respectively. The binding affinities of the compounds were calculated and ranked according to their highest negative values, which corresponded to their best binding affinities. A molecular docking study was performed using the AutoDock Vina to determine the protein’s interacting residues with specific ligands. Re-docking 6LU7 with its crystallographic inhibitor **N3** was performed to validate docking studies. 2D and 3D representations of protein-ligand complexes were visualized using Discovery Studio 2019 Client and Pymol Graphic Viewer software, respectively. (Nguyen et al., 2020; Arévalo and Amorim, 2022)

For LeDock, PDB format files of proteins and ligands were used as input files. The site and dimension of the grid box was identified according to the positive ligands in the crystallographic complex and are consistent with those in AutoDock Vina. The parameter of number of binding poses was considered to be 20. After the active pocket was placed and the parameter was set, LeDock calculations were performed for molecular docking. (Sun et al., 2021)

### Molecular docking data processing

The first step is to standardize the results obtained and establish a common docking assessment system. In our manuscript, 100 units correspond to the compound with the highest binding energy, and one unit corresponds to the compound with the smallest binding energy. For each molecular docking, the binding energies of each compound were normalized accordingly. The second step is to add the resulting normalized binding energies for each compound. The third step is to rank these compounds based on the total normalized binding energy. Consequently, compounds with false positives or weak activity will be ranked as low as possible in the list of normalized binding energies. The top 20 compounds in the list will be used as initial configurations for subsequent molecular dynamics simulations and the analysis of drug-likeness property, biological activity and ADMET.

### Molecular dynamics simulation procedures

Molecular dynamics (MD) simulation of 200 ns was carried out on the first 20 molecular docking systems screened. The GROMACS 5.1.4 software was used for the MD simulation studies, and the force field used was the GROMACS G54A7FF all-atom force field. Topology files for small molecule ligands were generated using the ATB server (http://atb.uq.edu.au/index.py). In a cube box with periodic boundary conditions, SPC model water molecules were added. Sodium or chloride counterions were added to neutralize each simulated system. Energy minimization was performed for 5,000 steps using steepest descent algorithm with a tolerance value of 100 kJ/mol/nm. Then, the M^pro^-ligand system was equilibrated with a position restriction of 500 ps (250,000 steps) using NVT and NPT ensembles, respectively, using V-rescale and Parrinello-Rahman method. The heating of the systems was gradually increased from 0 to 309.15 K, and the pressure of the systems was set to 1 atm for the NVT and NPT ensembles, respectively. Finally, production run for simulation was carried out at a constant temperature of 309.15 K and a pressure of 1 atm using V-rescale and Parrinello-Rahman algorithms, respectively. The LINCS method was used to constrain all bonds. Verlet scheme was used for the calculation of non-bonded interactions. The calculation method of long-range electrostatic interaction was PME, and the cut-off value of electrostatic action was set to 1.2 nm. Periodic boundary conditions (PBC) were used in all *x, y, z* directions. The final step of molecular dynamics simulation took 200 ns, with each step lasting 2 fs. The same molecular dynamics simulation was also performed for wild M^pro^ with the same parameters.

### Trajectory analysis of molecular dynamics simulation

The trajectory files generated by the MD simulation were used for the analysis of RMSD, SASA, H-bond numbers, and RMSF, and the analysis tools were derived from the methods provided by GROMACS 5.1.4. Hydrogen bond occupancy was also analyzed, using a method derived from a separate python script.

### Binding free energy and energy decomposition analysis

The binding free energy between protein and ligand was calculated using the MMPBSA method and the g_mmpbsa script. (Kumari et al., 2014) Molecular mechanical potential energy (electrostatic + van der Waals interaction energy) and solvation free energy (polar + nonpolar solvation energy) were used to calculate binding free energy, which were calculated at 500 ps intervals using 200 poses in the last 100 ns of the MD trajectory.

### Drug-likeness property

The drug-likeness property of the compounds screened by molecular docking was analyzed using DruLiTo software. (Joshi et al., 2021) According to the physicochemical properties of biologically active compounds, Lipinski’s rule was adopted for filtering it.

### Biological activity prediction

The biological activity of the compounds screened by molecular docking was predicted using the Molinspiration Cheminformatics online server (https://www.molinspiration.com) (Othman et al., 2020).

### ADMET

ADMET (absorption, distribution, metabolism, excretion, and toxicity) characteristic was predicted on selected biologically active ligands by pkCSM server (http://biosig.unimelb.edu.au/pkcsm/) (Pires et al., 2015). Combined with the molecular docking results, the SMILES structure files of these 20 compounds were retrieved from the ZINC20 database and used as input files for the pkCSM online tool.

## Results

### Molecular docking

**FIGURE 3:**
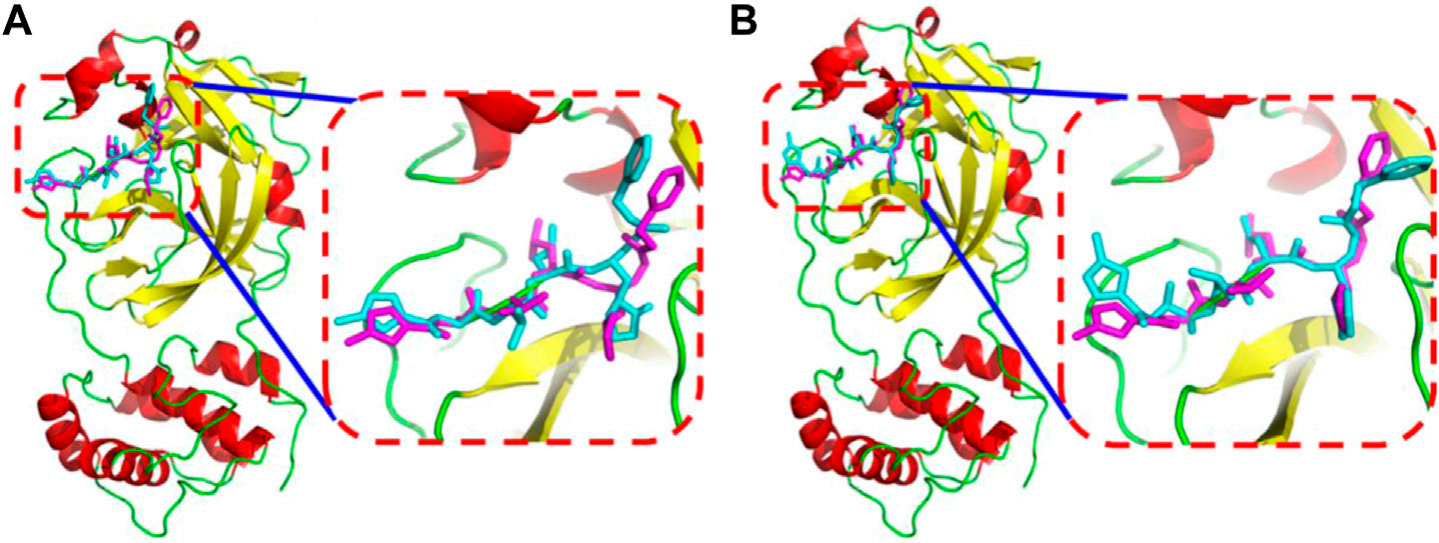
The superimposition between the **N3** in the crystal structure (magenta) and the **N3** from docked conformation (cyan) by AutoDock Vina **(A)** or by LeDock **(B)**.

**FIGURE 4:**
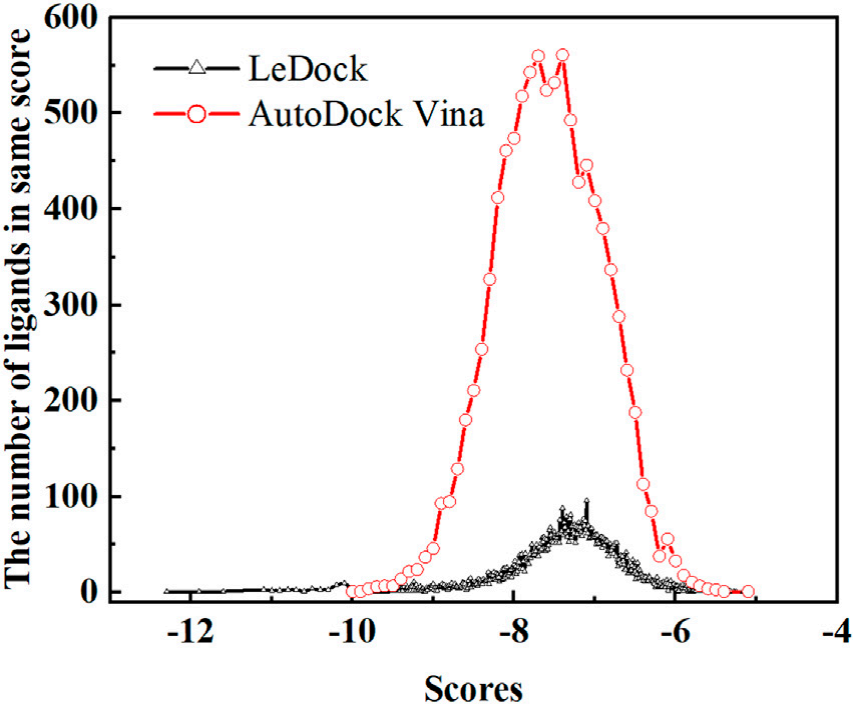
In LeDock and AutoDock Vina, the number of ligands with the same score.

Soy isoflavones are secondary metabolites produced during soybean growth and have many biological activities, among which genistein has the best activity. At present, many flavonoids have been studied against the SARS-CoV-2, but less research on the anti-COVID-19 based on genistein. (Santana et al., 2021; Alzaabi et al., 2022) In this work, firstly, the docking protocol had been validated by re-docking the coordinated ligand (**N3**) from the crystallographic structure of SARS-CoV-2 main protease (PDB ID: 6LU7) into the substrate-binding pocket. Then, LeDock and AutoDock Vina were used to conduct molecular docking studies on 9,614 genistein derivatives and the M^pro^ of SARS-CoV-2, respectively. The docking results showed a similarity between the ligand pose and coordinated pose (Figure 3) and the RMSD values didn’t exceed 2 Å, the binding affinity were −9.59 and −7.7 kcal/mol (Table 1) by LeDock and AutoDock Vina, respectively. The obtained results display that the docking protocol used in this study is reliable. The ZINC20 ID of top 20 ligands (the same score shows only one of the ligands) and their binding energies to the target are shown in Table 1. The first column represents the order of the molecular docking scoring results. The second and third columns represent the names of the ligands docked by LeDock and the corresponding scores, respectively. The fourth and fifth columns represent the names of the ligands docked with AutoDock Vina and the corresponding scores, respectively. It can be found from Table 1 that after docking with LeDock, lots of genistein derivatives had good affinity with the main protease, and the absolute value of the score exceeded 10. After docking with AutoDock Vina, it was found that some ligands also showed good binding force to the target protein, and some of them even exceeded the WHO-recommended drug Remdesivir [score by AutoDock Vina: −9.4 (Yalçın et al., 2021)] against COVID-19. Moreover, there are many genistein derivatives that score better than **N3**, both from LeDock and AutoDock Vina. However, comparing the results of the two molecular docking, it was obvious that the score ranking of the ligands in different molecular docking procedures was not consistent. For example, the highest scoring ligand **ZINC000253529553** in the LeDock ranked less than the top 20 in the AutoDock Vina, while in the AutoDock Vina, the highest scoring ligand **ZINC000072110832** was also not in the forefront of LeDock molecular docking. This may be caused by different algorithms adopted by different molecular docking software. In order to reduce the probability of false positive results, an effective method is to use a combination of different molecular docking methods. In addition, a statistical analysis on the docking scores of all ligands were also conducted, and found that the number of docking ligands showed a trend of more in the middle (about −7.5) and less on both sides with the distribution of docking scores (Figure 4), and the distribution trend of docking scores between the two molecules was highly consistent, which indicates that the two molecular docking results are related, and further correlation can be made.

**TABLE 1:** Top 20 results after 9,614 ligands docked with receptor by LeDock or AutoDock Vina.

Rank	Ligands docked by LeDock	LeDock score (kcal/mol)	Ligands docked by AutoDock vina	AutoDock vina score (kcal/mol)
1	**ZINC000253529553**	−12.3	**ZINC000072110832**	−10
2	**ZINC000253529555**	−11.9	**ZINC000096114211**	−9.9
3	**ZINC000253529554**	−11.6	**ZINC000072111338**	−9.8
4	**ZINC000253529552**	−11.1	**ZINC000085877721**	−9.7
5	**ZINC000253388752**	−11	**ZINC000096114537**	−9.6
6	**ZINC000085911544**	−10.9	**ZINC000169337465**	−9.5
7	**ZINC000169634432**	−10.8	**ZINC000002116527**	−9.4
8	**ZINC000085893593**	−10.7	**ZINC000059930660**	−9.3
9	**ZINC000253501057**	−10.6	**ZINC000085876477**	−9.2
10	**ZINC000059728719**	−10.5	**ZINC000014811805**	−9.1
11	**ZINC000197927867**	−10.4	**ZINC000001751803**	−9
12	**ZINC000238745543**	−10.3	**ZINC000002106564**	−8.9
13	**ZINC000082149236**	−10.2	**ZINC000002666274**	−8.8
14	**ZINC000085644998**	−10.1	**ZINC000002294283**	−8.7
15	**ZINC000150371810**	−10	**ZINC000000756489**	−8.6
16	**ZINC000067910683**	−9.98	**ZINC000002090387**	−8.5
17	**ZINC000150596901**	−9.92	**ZINC000005158973**	−8.4
18	**ZINC000072110371**	−9.91	**ZINC000001280533**	−8.3
19	**ZINC000150596856**	−9.9	**ZINC000001660868**	−8.2
20	**ZINC000253501059**	−9.89	**ZINC000000538127**	−8.1
Reference inhibitor	**N3**	−9.59	**N3**	−7.7

**FIGURE 5:**
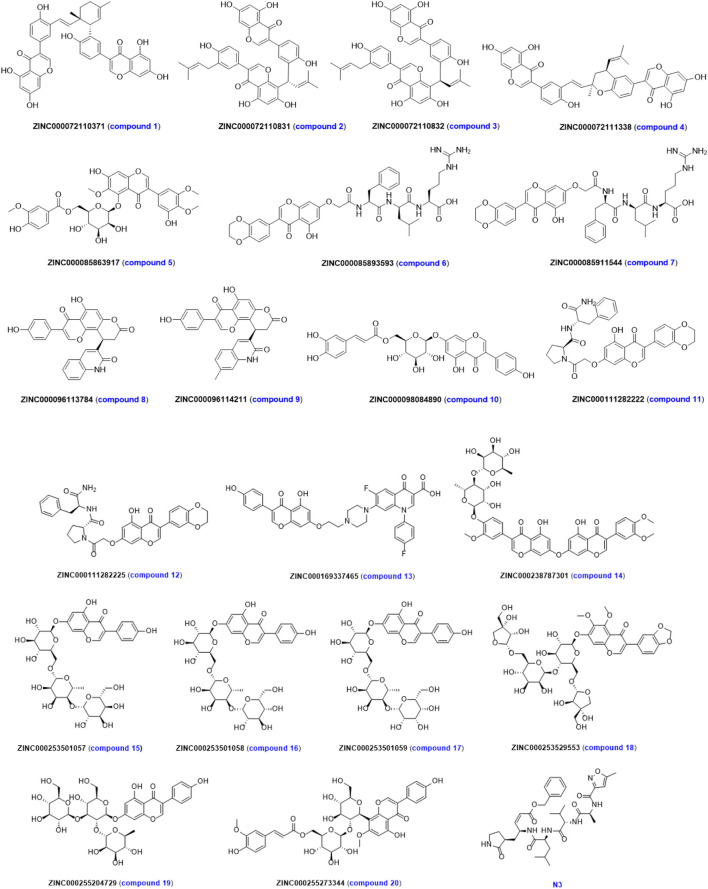
Structure of the top 20 compounds screened by normalized assessment.

In order to reduce the chance of false positives caused by single-molecule docking results, the results of two molecular docking were normalized. The evaluation results and molecular structures of the top 20 compounds after normalization are shown in Table 2 and Figure 5. The first column represents the ordering of the normalized scoring results. The second column represents the name of the ligand under that rank. The third and fourth columns represent the normalized scoring results corresponding to LeDock and AutoDock Vina in this ranking, respectively. The fifth column represents the total normalized scoring results. As can be seen in Table 2, the highest scoring molecules in LeDock or AutoDock Vina still ranked high in the normalized results. The screened 20 compounds with high normalized binding energy had more heteroatoms such as nitrogen, and oxygen atoms in the structure, and the molecular structure was rich in hydroxyl and amino groups. More importantly, the chirality of the molecule has a significant impact on the strength of the binding force, such as **ZINC000072110831** (compound **2**) and **ZINC000072110832** (compound **3**) due to the difference in the cis-trans isomerism of the vinyl group in the molecular structure, the normalized affinity of the two compounds to the target protein was different. In addition, it was also found that molecules with more sugars such as **ZINC000253501057** (compound **15**), **ZINC000253501058** (compound **16**), and **ZINC000253501059** (compound **17**) did not have the highest normalized affinity. This may be due to the increased hydrophilicity of ligand molecules, resulting in weakened binding to hydrophobic protein sites.

**TABLE 2:** ZINC20 ID and normalized scores of the top 20 genistein derivatives after normalization of molecular docking results.

Rank	Ligands	LeDock	AutoDock vina	Sum
1	**ZINC000072110832**	53.7289	**100**	**153.7289**
2	**ZINC000085911544**	80.777	71.72	152.497
3	**ZINC000255273344**	79.404	69.7	149.104
4	**ZINC000072111338**	49.7472	95.96	145.7072
5	**ZINC000096114211**	47.6877	97.98	145.6677
6	**ZINC000072110371**	67.1843	77.78	144.9643
7	**ZINC000111282222**	58.5344	85.86	144.3944
8	**ZINC000255204729**	82.15	61.62	143.77
9	**ZINC000253529553**	**100**	43.44	**143.44**
10	**ZINC000072110831**	62.7907	79.8	142.5907
11	**ZINC000098084890**	62.6534	79.8	142.4534
12	**ZINC000096113784**	52.9051	87.88	140.7851
13	**ZINC000253501059**	66.9097	73.74	140.6497
14	**ZINC000169337465**	50.2964	89.9	140.1964
15	**ZINC000085893593**	78.031	61.62	139.651
16	**ZINC000085863917**	61.4177	77.78	139.1977
17	**ZINC000253501058**	75.285	63.64	138.925
18	**ZINC000111282225**	52.9051	85.86	138.7651
19	**ZINC000238787301**	60.7312	77.78	138.5112
20	**ZINC000253501057**	76.658	61.62	138.278
Reference inhibitor	**N3**	62.7907	53.54	116.3307

**FIGURE 6:**
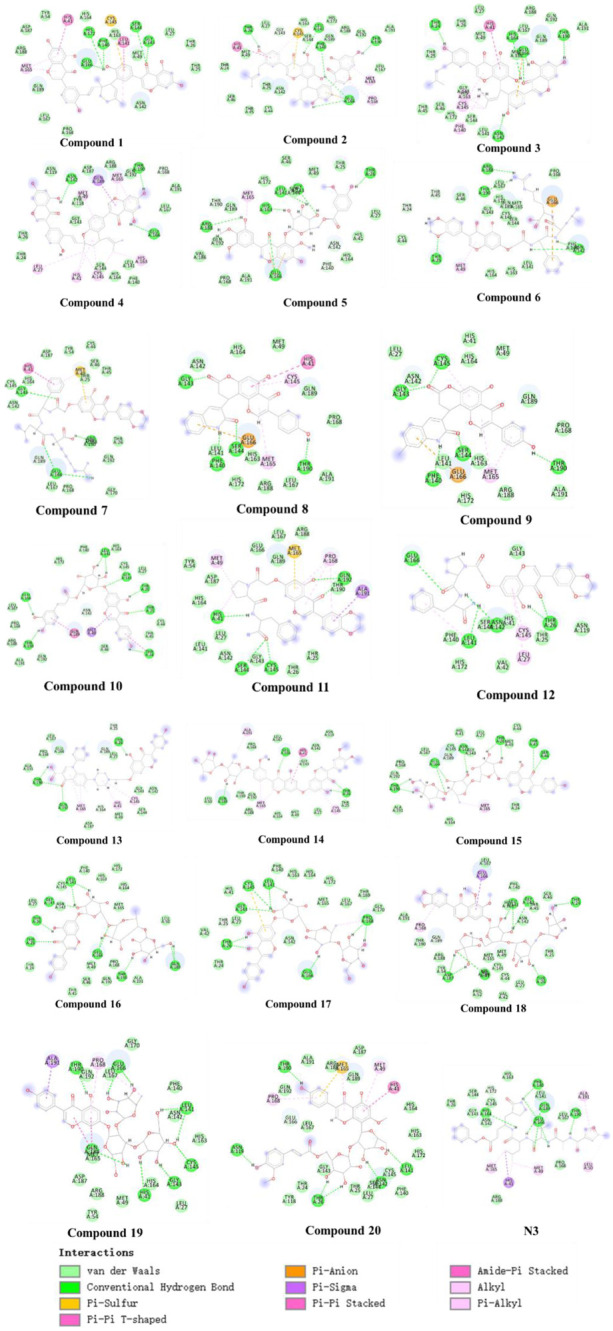
2D presentations of interactions of the top 20 genistein derivatives and **N3** with SARS-CoV-2 main protease, respectively.

Further, we analyzed the 2D and 3D docking maps of these 20 compounds with the main protease (Figure 6 and Supplementary Figure S1). It can be seen from Supplementary Figure S1 that most of the compounds, like **N3**, could fit well with the active pocket of the main protease after molecular docking. Due to the differences in their own structures and sizes, some compounds show a significantly different conformation from **N3** when binding to the main protease. For example, when compound **1** binds to the protease, the molecular conformation tends to cohesion. However, due to the large molecular size of compound **14**, some groups are exposed outside the active pocket. In terms of the mechanism of action (Figure 6), it was found that these compounds could interact with the main protease by forming hydrogen bonds, van der Waals forces, π-π stacking, etc., but compared with **N3**, the interacting groups are different. For example, the carbonyl, hydroxyl and amide bonds of the flavonoid fragment in preferred compound **11** could form multiple hydrogen bonds with HIS41, SER144, CYS145, and GLN192, respectively, but **N3** mainly formed hydrogen bonds with residues such as PHE140, HIS164, GLU166, GLN189, and THR190 of the main protease. However, residues such as HIS164, GLU166, GLN189, and THR190 could interact with compound **11** in van der Waals. Similarly, residues such as HIS41, SER144, and CYS145 could also interact with **N3** in other non-bonding ways. These results suggest that these compounds share the active pocket with **N3**, the mode of action and the group of action are different.

### Molecular dynamics simulation

**FIGURE 7:**
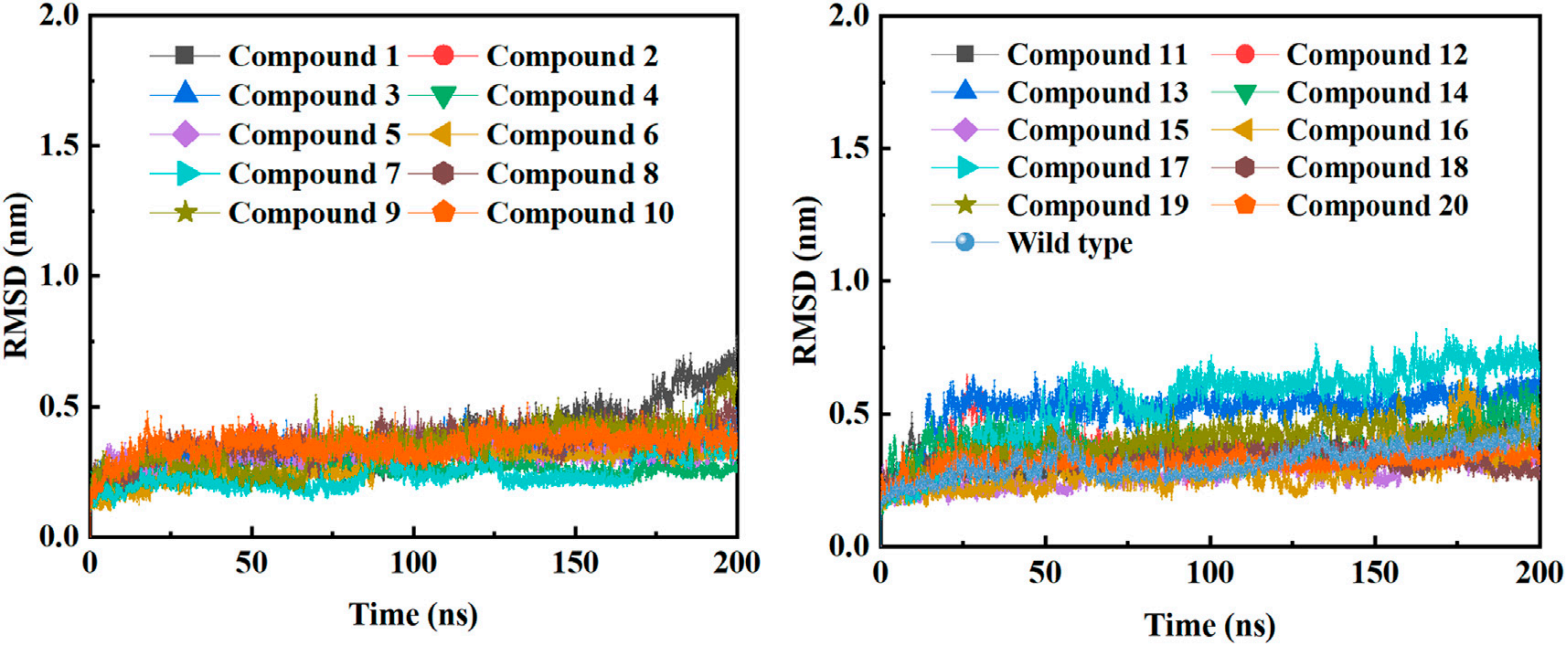
RMSD of backbone C-alpha atoms of COVID-19 M^pro^ relative to the starting complexes at different ligand systems or wild M^pro^ during 200 ns MD run.

**FIGURE 8:**
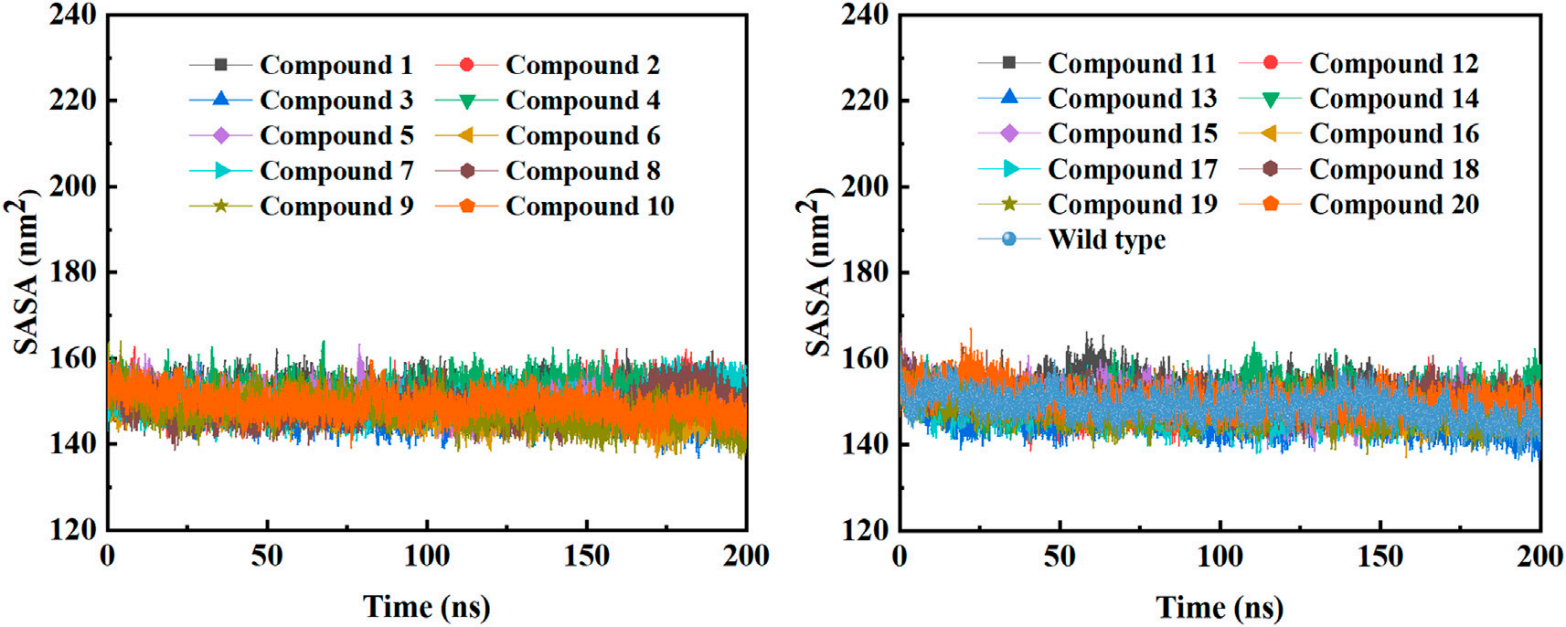
The SASA plots of COVID-19 M^pro^ in a MD simulation system containing different ligands or wild M^pro^.

**FIGURE 9:**
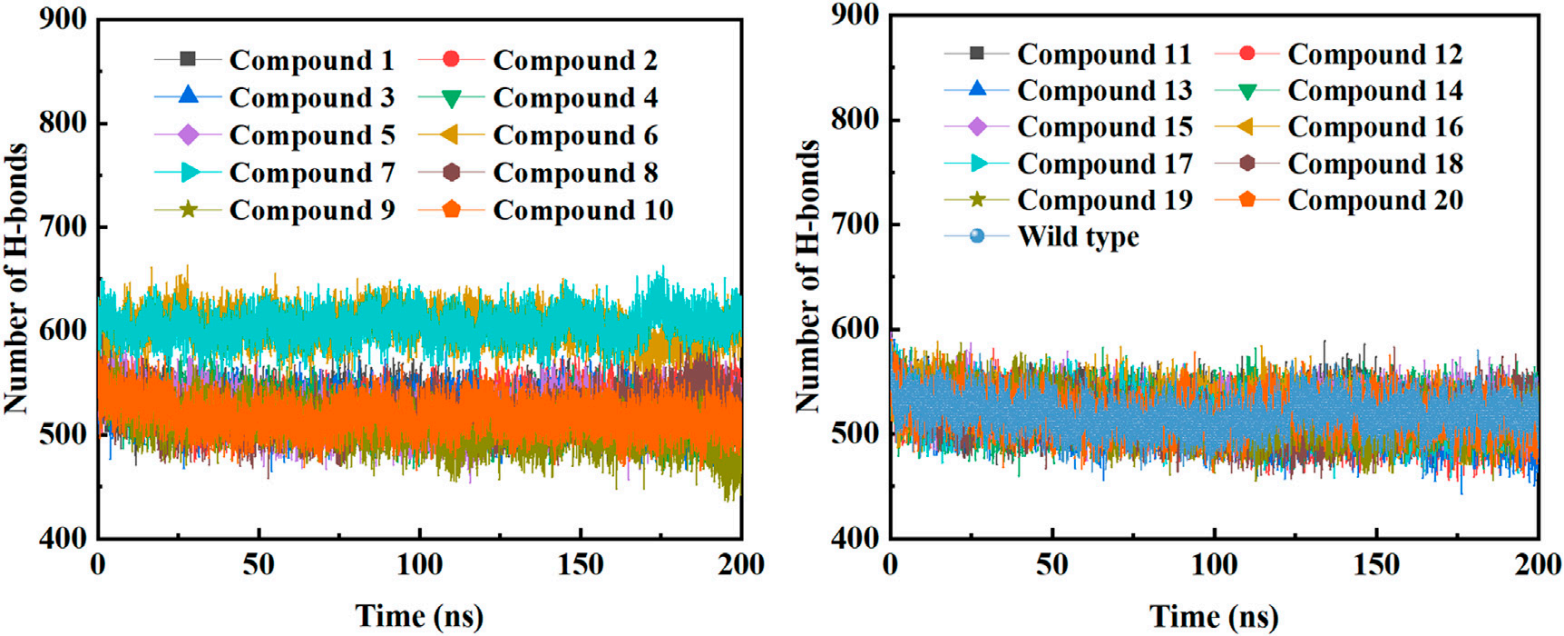
The changes in the number of H-bonds during the MD trajectory of different M^pro^-ligand system or wild M^pro^.

**FIGURE 10:**
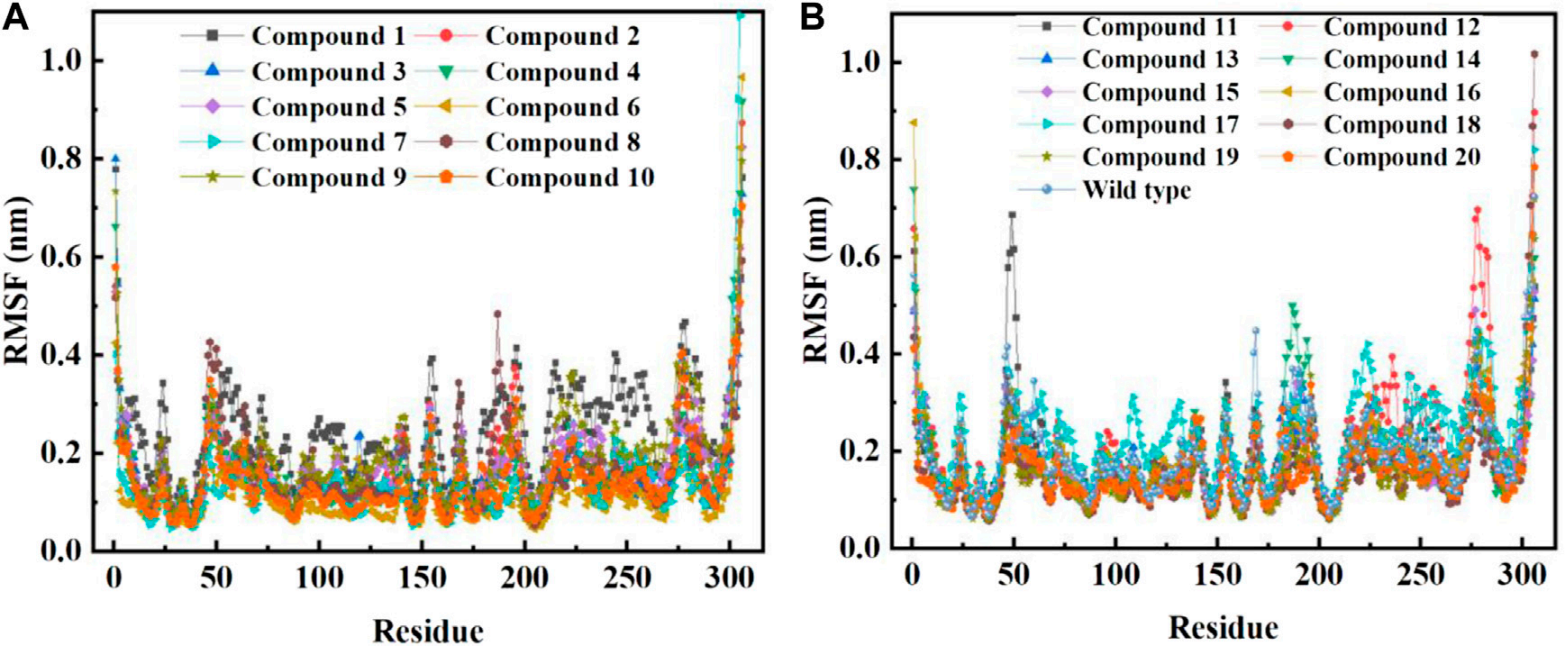
RMSF per residue of backbone C-alpha atoms of COVID-19 M^pro^ relative to the starting complexes at different ligand systems or wild M^pro^ during 200 ns MD run.

In order to further study the interaction stability of the 20 compounds screened by molecular docking and the 2019-nCoV target, MD simulation was used to study the interaction between these 20 compounds screened and protein receptor. The root mean square deviation (RMSD) value over time is often used to check whether a simulation system has reached stability. Figure 7 shows the RMSD values of main protease as a function of time at different simulation system. The RMSD values for the M^pro^ shown on the y-axis in Figure 7 are based on all backbone C-alpha atoms relative to the corresponding starting structures of all trajectories for the simulated M^pro^-ligand system and wild M^pro^. The RMSD values of most systems fluctuated very little after 25 ns, indicating that the vast majority of the protein-ligand complexes and wild M^pro^ attained a stable conformation during the simulation runs. In addition to the RMSD, solvent accessible surface area (SASA) and the number of H-bonds are also two important thermodynamic parameters. (Ogunyemi et al., 2022) SASA refers to the level of the solvent accessibility surface of the proteins. H-bonds reflects the interaction between the complex and the solvent throughout the simulation. It can be seen from Figure 8 that for all complex systems and wild M^pro^, the SASA values remained between 140 and 160 nm^2^ throughout the simulation process, with no large fluctuations, which indicates that the interaction between the main protease and the surrounding solvent is less affected by the ligand throughout the simulation. As for the number of H-bonds (Figure 9), the simulation results for all complex systems and wild M^pro^ are similar to SASA except compound **6** and compound **7**. Although the number of hydrogen bonds of compound **6** and compound **7** is higher than that of other compounds, the two simulation systems are relatively stable throughout the simulation process. These results show that all simulated systems reach an equilibrium state. In addition, the root mean square fluctuation (RMSF) diagram of each residue of side chain and main chain of the protein on the MD trajectory was also calculated, as shown in Figure 10. The RMSF values of most residues in all simulated complexes were within 3.0 Å, and the RMSF values of the amino acid residues adjacent to the head and tail were larger, which may be due to the fact that these residues are less bound by weak intramolecular interactions and their conformation in the solvent big change. The hydrogen bond occupancy during the interaction between ligands and main protease was also analyzed and the results are shown in Table 3. The first column represents the name of the compound. The second column indicates that atoms of this compound form hydrogen bonds with residues of the main protease. The third column indicates the probability of this hydrogen bond occurring. Plenty of ligands were able to form stable hydrogen bonds with protein receptor (the hydrogen bond occupancy was over 50%) such as compounds **2**, **7**, **9**, **10**, **12**, **13**, **18**, and **19**, especially, compound **7** could form multiple stable hydrogen bonds with amino acid residues of main protease, such as GLU166 and ARG188, etc. From the two-dimensional diagram of molecular docking, it can be seen that the guanidine and carboxyl hydrogen atoms and carbonyl oxygen atoms of main protease were mainly responsible for the interaction with compound **7**. Although other ligands were also capable of forming hydrogen bonds with protein receptor, but hydrogen bond occupancy did not exceed 50%, indicating that the interaction between other ligands and the target protein may be dominated by other weak interactions such as van der Waals.

**TABLE 3:** Hydrogen bond occupancy of amino acid residues participating in H-bonding with the top 20 compounds, during entire MD simulation (only show more than 50% of results).

Compounds	Donor-acceptor	Occupancy (%)
**2**	**2**(H)-44CYS(O)	59.8
**7**	**7**(H46)-188ARG (O)	95.5
	**7**(H44)-166GLU (O)	55.4
	**7**(H42)-166GLU (O)	96.8
	166GLU (H)-**7**(O9)	98.2
	41HIS(HE2)-**7**(O2)	86.6
**9**	166GLU(H)-**9**(O)	52.4
**10**	**10**(H)-164HIS(O)	68.0
**12**	166GLU(H)-**12**(O)	61.6
**13**	**13**(H)-166GLU(OE2)	52.7
**18**	**18**(H34)-26THR(O)	58.8
	**18**(H32)-26THR(O)	62.7
	**18**(H16)-187ASP(O)	51.5
**19**	40ARG (HE)-**19**(O)	54.7

**FIGURE 11:**
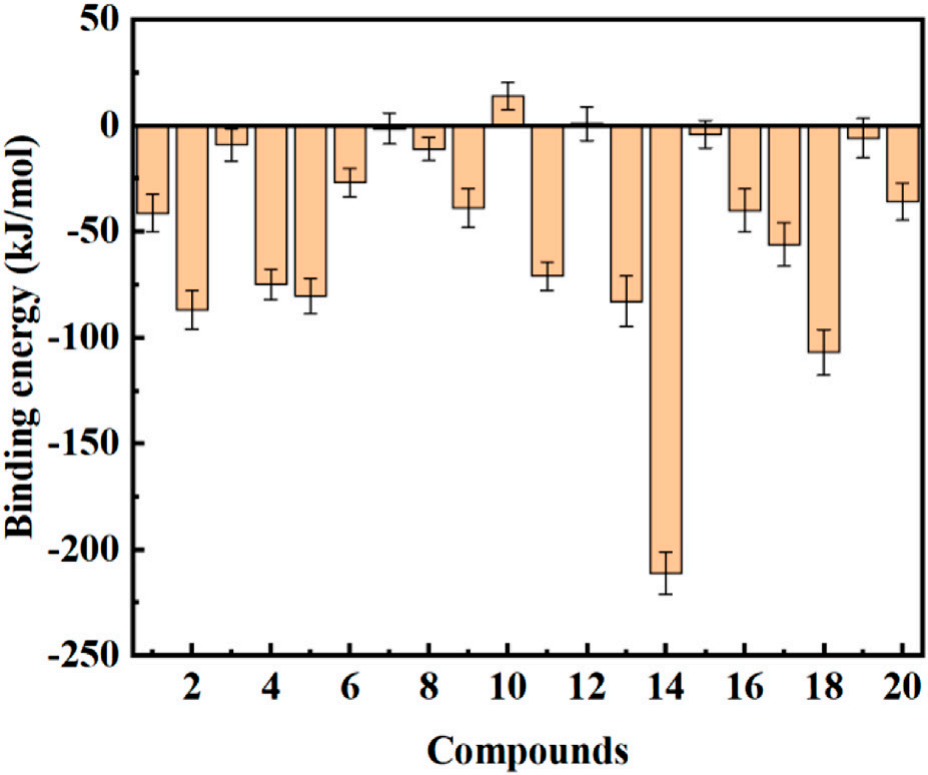
Binding free energy calculation between the COVID-19 M^pro^ and the top 20 compounds screened by normalized assessment.

Most molecular docking software estimates binding energies through force field calculations guided by quantum mechanics. However, precise binding energies can only be determined by ab initio methods such as DFT and molecular dynamics simulations. To further study the interaction between ligands and protein receptor, the binding free energies of ligands and protein receptor were calculated using g_mmpbsa, and the energy contributions were also decomposed. The results are shown in Figure 11 and Table 4. It can be seen from Figure 11 that compounds **2**, **4**, **5**, **11**, **13**, **14**, **17**, and **18** had a good binding effect with the target, and the binding free energy was less than −50 kJ/mol, which is very worth for further research. Especially for compound **14**, its binding effect with the target was far more than other compounds, and the binding free energy reached −211.032 kJ/mol. In addition, it was also found that the binding free energies of compounds **10** and **12** were positive, which indicates that these two compounds cannot spontaneously bind to target protein. These results suggest that after rapid molecular docking screening, accurate molecular dynamics simulations are necessary for further drug screening.

**TABLE 4:** Binding free energy calculation between the COVID-19 M^pro^ and the top 20 compounds screened by normalized assessment.

Compounds	van der Waal energy (kJ/mol)	Electrostatic energy (kJ/mol)	Polar solvation energy (kJ/mol)	Non-polar solvation energy (kJ/mol)	Binding energy (kJ/mol)
**1**	−67.704±12.902	−6.877±1.369	40.206±7.732	−6.507±1.224	−41.296±8.719
**2**	−123.140±12.437	−29.106±2.895	78.140±9.413	−13.024±1.351	−87.003±8.990
**3**	−33.309±8.334	−5.747±1.543	33.890±6.490	−4.000±0.877	−9.176±7.658
**4**	−141.762±11.928	−12.761±1.243	94.609±8.510	−15.360±1.361	−74.980±7.229
**5**	−133.551±12.604	−35.267±3.612	102.329±10.639	−13.862±1.244	−80.485±8.387
**6**	−109.139±8.933	−122.599±10.076	216.335±16.066	−11.761±0.945	−26.968±6.645
**7**	−47.615±10.542	−21.740±4.887	72.780±12.454	−4.853±1.113	−1.506±7.276
**8**	−21.625±5.247	−2.904±0.821	16.000±5.472	−2.709±0.630	−11.051±5.494
**9**	−77.130±10.087	−11.079±1.578	56.507±6.669	−7.055±1.096	−38.945±9.180
**10**	−3.615±2.593	−0.783±0.449	18.501±6.511	−0.576±0.498	13.741±6.539
**11**	−163.660±11.679	−32.155±3.069	141.999±10.354	−17.381±1.213	−71.057±6.760
**12**	−37.051±8.789	−6.649±1.649	47.399±7.566	−3.758±0.912	0.842±7.932
**13**	−122.643±14.459	−20.831±2.517	72.814±7.018	−11.917±1.339	−83.000±11.921
**14**	−312.314±12.731	−30.476±1.697	163.468±5.872	−31.887±1.200	−211.032±10.007
**15**	−36.981±9.519	−10.191±2.611	46.881±8.765	−3.789±1.041	−4.175±6.517
**16**	−81.179±11.917	−17.549±2.743	66.346±7.666	−7.694±1.223	−40.077±10.147
**17**	−100.545±13.438	−29.458±4.038	84.772±10.756	−10.302±1.307	−56.059±10.199
**18**	−177.868±15.707	−46.486±4.288	133.605±11.676	−17.249±1.463	−107.000±10.752
**19**	−40.956±9.775	−11.759±2.872	51.755±9.104	−4.142±0.943	−5.805±9.348
**20**	−73.897±12.278	−11.723±2.039	57.970±8.549	−8.249±1.385	−35.925±8.681

From the energy decomposition results (Table 4), it can be seen that the binding free energy can be divided into four components: van der Waals interaction energy, electrostatic energy, polar solvation energy, and non-polar solvation energy. For all systems, the van der Waals interaction and the electrostatic interaction are beneficial to the binding of the ligand to the receptor, and the van der Waals interaction is stronger than the electrostatic interaction (except for compound **6**). Polar solvation energies and non-polar solvation energies have opposite contributions to the binding free energy. Although non-polar solvation energies promote the binding of ligands to protein receptors, they account for a small proportion, while polar solvation energies are not conducive to the binding of ligands to receptors, but play a much greater role than non-polar solvation energies.

### Drug-likeness analysis

Based on the results of the normalized assessment, the drug-likeness of the top 20 compounds screened was analyzed (Table 5). The Lipinski rule was adopted to evaluate the drug-like properties of these ligands, and it is generally considered that the chemical molecules with LogP≤5, HBD≤5, HBA≤10, MW ≤ 500, and nRB≤10 passed the drug-like property evaluation. (Gorla et al., 2021) These properties have a crucial impact on the interaction between chemical molecules and their targets, as well as the absorption, distribution, metabolism, excretion, and toxicity of drugs. Therefore, compounds with the above characteristics have the potential to become drug candidates. Because the evaluation of drug-like properties of drugs is based on the physicochemical properties of chemical molecules, Lipinski’s five rules are the preliminary criteria for evaluating the drug-like properties of ideal drug structures. Although the molecular weight of all compounds except compounds **8** and **9** exceed 500, it does not mean that these compounds are not possible to become drugs. It can be solved the problems such as permeability caused by large molecular weight compounds through advanced preparation technology. Interestingly, it can be seen from Table 5 that logp values of all compounds are less than 5, indicating that all compounds have relatively ideal oil-water distribution coefficients. The number of hydrogen bonds formed between the compound and the target will affect their interaction. Compounds **1**∼**4**, **8** and **9** conform to the rule that the number of hydrogen bond acceptors is less than 10 and compounds **10**∼**13** are close to the rule, but other compounds do not conform to this rule. Compounds **4**, **8**, **9**, **11**∼**13** fit the rule of hydrogen bond donor number. In general, the number of rotatable bonds indirectly affects other properties of compounds. In all compounds, except compounds **6**, **7**, and **18**, which seriously deviate from the rule of less than 10, the number of rotatable bonds in other compounds is either less than or close to 10. In this work, compounds **8** and **9** followed these five rules, compounds **1**∼**4**, **10**∼**13, 15**∼**17**, and **19** followed 2–4 of them, and the other compounds followed fewer rules. However, a drug does not have to follow all the rules to become a potential drug candidate, especially in recent years, with the development of pharmaceutical technology, the use of new technologies can make up for the lack of a certain property of the chemical molecule. The study by Mudliar et al. found that the highly active remdesivir only followed the two rules of the DruLiTo study. (Murugesan et al., 2021) These results suggest that the above-mentioned compounds following the rule of two or more deserve further development.

**TABLE 5:** Analysis of drug-like properties of the top 20 compounds screened by normalized assessment.

Compounds	MW	logP	HBA	HBD	nRB
**1**	672.2	4.759	10	6	5
**2**	674.22	4.932	10	6	7
**3**	674.22	4.932	10	6	7
**4**	672.2	4.39	10	5	5
**5**	672.17	1.356	16	6	11
**6**	786.32	0.609	17	8	22
**7**	786.32	0.609	17	8	22
**8**	467.1	0.779	8	3	2
**9**	481.12	1.171	8	3	2
**10**	594.14	1.016	13	7	8
**11**	613.21	1.239	12	3	11
**12**	613.21	1.239	12	3	11
**13**	681.19	3.46	11	3	8
**14**	888.25	1.721	19	7	11
**15**	740.22	−2.634	19	11	9
**16**	740.22	−2.634	19	11	9
**17**	740.22	−2.634	19	11	9
**18**	930.26	−3.425	25	11	15
**19**	740.22	−2.634	19	11	9
**20**	784.22	−0.847	18	9	12

MW, Molecular weight; logP, Partition coefficient in oil to water; HBA, Number of hydrogen bond acceptors; HBD, Number of hydrogen bond donors; nRB, Number of rotatable bonds.

### Biological activity prediction analysis

In this work, the 20 genistein derivatives derived from the molecular docking were utilized for a bioactivity score analysis of different targets, including GPCR ligands, ion channel modulators, nuclear receptor ligands and enzyme inhibitors (protease, kinase, etc.). Generally, compounds with scores greater than 0 are considered highly active compounds, compounds with scores between −0.5 and 0 are moderately active compounds, and compounds with scores below −0.5 are inactive compounds. (Murugesan et al., 2021) It can be seen from Table 6 that compounds **8** and **9**, and compounds **11** and **12** acted on nuclear receptors and proteases, respectively, and the corresponding scores were all higher than 0. In addition, the aforementioned compounds **8**, **9**, **11**, **12**, and **10** were also found to exhibit moderate activity against other targets (such as GPCR, Kinase), with corresponding scores ranging from −0.5 to 0. The other compounds showed little activity against all targets. From the prediction results of biological activity score, it can be seen that the key compounds **11** and **12** can be further developed as COVID-19 M^pro^ inhibitors.

**TABLE 6:** Biological activity prediction of the top 20 compounds screened by normalized assessment.

Compounds	GPCR ligand	Ion channel modulator	Kinase inhibitor	Nuclear receptor ligand	Protease inhibitor	Enzyme inhibitor
**1**	−0.666,	−1.701	−1.136	−0.803	-0.657	-0.802
**2**	−0.667	−1.724	−1.084	−0.818	-0.641	-0.841
**3**	−0.667	−1.724	−1.084	−0.818	-0.641	-0.841
**4**	−0.642	−1.704	−1.135	−0.787	-0.576	-0.772
**5**	−0.556	−1.397	−0.865	−0.774	-0.539	-0.58
**6**	−1.523	−2.923	−2.532	−2.611	-0.936	-2.036
**7**	−1.659	−3.028	−2.567	−2.556	-1.094	-2.102
**8**	−0.095	−0.444	−0.128	0.084	-0.455	0.082
**9**	−0.128	−0.495	−0.151	0.068	-0.477	0.043
**10**	−0.138	−0.782	−0.329	−0.033	-0.254	-0.009
**11**	−0.046	−0.922	−0.395	−0.311	0.049	-0.308
**12**	−0.046	−0.922	−0.395	−0.311	0.049	-0.308
**13**	−0.707	−1.639	−0.963	−0.914	-0.592	-0.896
**14**	−3.166	−3.653	−3.532	−3.536	-2.722	-3.244
**15**	−0.896	−2.012	−1.378	−1.431	-0.734	-1.009
**16**	−0.896	−2.012	−1.378	−1.431	-0.734	-1.009
**17**	−0.896	−2.012	−1.378	−1.431	-0.734	-1.009
**18**	−3.221	−3.661	−3.568	−3.619	-2.874	-3.25
**19**	−0.908	−2.044	−1.42	−1.401	-0.718	-1.026
**20**	−1.541	−2.972	−2.242	−2.26	-1.29	-1.74

### ADMET analysis

The pharmacokinetic properties of drug candidates include absorption, distribution, metabolism, excretion and toxicity, namely ADMET. (Han et al., 2019) ADMET analysis is very helpful in the discovery phase of new drugs. Drug candidates that have passed ADMET analysis are significantly less likely to fail in subsequent clinical trials. ADMET analysis was performed on the 20 compounds screened by standardized assessment in this research, as shown in Tables 7, 8. In the new drug discovery process, absorption parameters mainly include water solubility, GI (gastrointestinal) absorption, skin and Caco2 permeability, etc. When the GI value is >30%, it means that the drug molecule has good absorption and most of the compounds have good absorption. The gastrointestinal absorption of compounds **8** and **9** could reach 100%, but the GI value of compounds **6**, **7**, **15**, **16**, **17**, **18**, and **19** were all less than 30%, especially the GI values of compounds **15**, **16**, **17**, and **18** were 0, indicating that these compounds are barely absorbed. Skin penetration less than −2.5 is considered low penetration, and all of these 20 genistein derivatives exerted acceptable skin penetration. Caco2 permeability was low (<0.9) for all compounds except compound **5** (0.934). Another important factor in absorption analysis is the prediction of P-glycoprotein non-substrate drug candidates. Except for compounds **4** and **9**, all compounds were substrates of P-glycoprotein. The distribution of drug molecules was investigated using VDss (distribution volume), CNS (central nervous system) and BBB (blood-brain barrier) permeability. LogVDss greater than 0.45 is considered to have a higher volume of distribution, and less than −0.15 is considered to have a small volume of distribution. Among these 20 compounds, only compounds **6**, **7**, **10∼12**, and **18∼20** had a moderate volume of distribution. For the permeability of the blood-brain barrier, when the logBBB value is less than -1, it is considered difficult for drug molecules to pass through the blood-brain barrier. All compounds had poor blood-brain barrier permeability, and most had poor CNS permeability (logCNS < −3).

**TABLE 7:** Absorption and distribution prediction of the top 20 compounds screened by normalized assessment.

Ligands	Absorption					Distribution		
	Water solubility	Caco2 permeability	Intestinal absorption	Skin permeability	P-glycoprotein substrate	VDss	BBB permeability	CNS permeability
**1**	−2.896	−0.042	92.328	−2.735	Yes	−1.436	−1.925	−2.932
**2**	−2.892	−0.535	91.568	−2.735	Yes	−0.85	−2.009	−2.892
**3**	−2.892	−0.535	91.568	−2.735	Yes	−0.85	−2.009	−2.892
**4**	−3.001	0.14	92.606	−2.735	No	−1.496	−1.89	−2.793
**5**	−2.909	0.934	46.051	−2.735	Yes	−0.227	−2.224	−5.092
**6**	−2.892	−0.177	18.379	−2.735	Yes	0.01	−2.188	−4.738
**7**	−2.892	−0.177	18.379	−2.735	Yes	0.01	−2.188	−4.738
**8**	−3.416	0.064	100	−2.735	Yes	−1.517	−1.189	−3.153
**9**	−3.416	0.708	100	−2.735	No	−1.273	−1.241	−3.253
**10**	−3.271	0.567	57.312	−2.735	Yes	−0.015	−1.882	−4.391
**11**	−3.307	0.328	78.473	−2.735	Yes	0.048	−1.732	−3.882
**12**	−3.771	0.413	95.896	−2.735	Yes	0.307	−1.327	−3.985
**13**	−2.958	0.359	77.864	−2.735	Yes	−0.946	−1.866	−3.514
**14**	−2.893	0.773	49.936	−2.735	Yes	−0.525	−2.578	−4.962
**15**	−2.883	−0.345	0	−2.735	Yes	−0.292	−2.011	−6.203
**16**	−2.883	−0.345	0	−2.735	Yes	−0.292	−2.011	−6.203
**17**	−2.883	−0.345	0	−2.735	Yes	−0.292	−2.011	−6.203
**18**	−2.89	−0.504	0	−2.735	Yes	−0.13	−2.554	−7.055
**19**	−2.884	−0.396	6.15	−2.735	Yes	−0.136	−2.305	−6.289
**20**	−2.904	−0.072	48.815	−2.735	Yes	−0.03	−2.259	−5.606
**Unit**	(log mol/L)	(log Papp in 10^–6^ cm/s)	(% Absorbed)	(log Kp)	(Yes/No)	(log L/kg)	(log BBB)	(log CNS)

**TABLE 8:** Metabolism, excretion and toxicity prediction of the top 20 compounds screened by normalized assessment.

Ligands	Metabolism			Excretion			Toxicity			
	CYP2D6 substrate	CYP3A4 substrate	CYP1A2 inhibitior	CYP2C19 inhibitior	Total clearance	AMES toxicity	hERG1 inhibitor	LD50	LOAEL	Skin sensitization
**1**	No	No	No	No	0.388	No	No	2.479	2.343	No
**2**	No	No	No	No	0.405	No	No	2.474	3.331	No
**3**	No	No	No	No	0.405	No	No	2.474	3.331	No
**4**	No	Yes	No	Yes	−1.469	No	No	2.432	1.713	No
**5**	No	No	No	No	0.863	No	No	2.638	5.321	No
**6**	No	Yes	No	No	−0.39	No	No	2.482	6.246	No
**7**	No	Yes	No	No	−0.39	No	No	2.482	6.246	No
**8**	No	Yes	No	Yes	0.382	No	No	2.881	1.498	No
**9**	No	Yes	Yes	No	0.458	No	No	2.859	1.505	No
**10**	No	No	No	No	−0.068	No	No	2.767	4.378	No
**11**	No	Yes	No	No	0.568	No	No	3.292	3.46	No
**12**	No	Yes	No	No	−0.093	No	No	2.608	3.027	No
**13**	No	Yes	No	No	−0.313	No	No	2.601	1.443	No
**14**	No	No	No	No	−1.278	No	No	2.502	5.828	No
**15**	No	No	No	No	0.396	No	No	2.371	7.265	No
**16**	No	No	No	No	0.396	No	No	2.371	7.265	No
**17**	No	No	No	No	0.396	No	No	2.371	7.265	No
**18**	No	No	No	No	−0.081	No	No	2.478	9.114	No
**19**	No	No	No	No	0.377	No	No	2.413	7.99	No
**20**	No	No	No	No	−0.025	No	No	2.536	5.885	No
**Unit**	(Yes/No)	(Yes/No)	(Yes/No)	(Yes/No)	(log ml/min/kg)	(Yes/No)	(Yes/No)	(mol/kg)	(log mg/kg_bw/day)	(Yes/No)

Cytochrome p450 plays a fundamental role in the metabolism of drugs in the liver system, and there are many subtypes, such as CYP2D6, CYP3A4, CYP1A2, and CYP2C19. The results of metabolic scoring shows that all the compounds couldn’t act on CYP2D6, and compounds **1**∼**3**, **5**, **10**, **14∼20** had no inhibitory on CYP3A4. In addition, except for compounds **4**, **8**, and **9**, other compounds had no effect on CYP1A2 and CYP2C19. The total clearance of the drug is decided by a combination of hepatic and renal clearance, which is defined by the rate of elimination of the drug from the body per unit time. The predicted results suggest that the excretion range of the candidate drug was −1.469 to 0.863. Among them, compounds **4**, **6**, **7**, **10**, **12**∼**14**, **18**, and **20** showed low drug clearance rate (<0.1), while other compounds showed moderate drug clearance rate (>0.1 to <1). In the process of drug development, toxicity is a non-negligible criterion. The selected drug candidates not only require high activity, but also have low toxicity. Therefore, toxicity plays an important role in selecting the most suitable drug candidates. All 20 compounds screened by molecular docking had no effect on skin. Inhibition of potassium channels encoded by human ether-a-go-go-related gene (hERG) is one of the reasons for drug-induced cardiotoxicity. Therefore, it is necessary to analyze the hERG-inhibiting ability of newly discovered drug molecules. All compounds had no inhibitory effect on hERG1. None of the drug candidates exhibited AMES (assay of the ability of a chemical compound to induce mutations in DNA) toxicity. The LD50 (median lethal dose) and the LOAEL (lowest observed adverse reaction level) of the candidate drugs were also predicted on the pkCSM online server, and the predicted results were shown in Table 8. All compounds showed low toxicity, especially compound **11**, which had higher LD50 value than other compounds and had the least toxicity.

## Discussion

Recently, the fatality rate of the COVID-19 has been significantly reduced, but for patients with other chronic diseases, the impact of the virus on life and health is still huge. Therefore, the discovery of anti-SARS-CoV-2 drugs is still important. As a natural product in soybean, isoflavones have a wide range of biological activities, especially genistein, an important component of isoflavones, has received extensive attention. Therefore, in this work, a series of computer-aided drug virtual screening studies were carried out with genistein, as the lead compound and the main protease of the SARS-CoV-2 as the target.

Considering that a single molecular docking method is prone to false positives, we used different methods to conduct molecular docking studies on ligands and receptors, and normalized the docking results. It was found that the molecules with the highest scores in either LeDock or AutoDock Vina still ranked high in the normalized results. The screened 20 compounds with high normalized binding energy contained a large number of heteroatoms in their structures, such as hydroxyl and amino groups. More importantly, the chirality of the molecule also has a significant impact on the strength of the binding force. For example, compounds **2** and **3** had very different normalized affinities to target protein due to the difference in the cis-trans isomer structure of the vinyl group. Furthermore, molecules containing more sugars such as compounds **15**, **16**, and **17** did not have the highest normalized affinity. These results indicate that heteroatoms should be considered in the design of non-covalent inhibitor drugs. However, due to the high polarity and strong hydrophilicity of heteroatoms, too many heteroatom-containing groups may sometimes hinder the compounds from acting on drug targets. Therefore, when designing new drugs, it is very necessary to reasonably increase or decrease the polar groups of heteroatoms on the basis of a certain configuration (cis or trans, R or S configuration). Most of the compounds, like **N3**, could fit well with the active pocket of the main protease after molecular docking. In terms of the mechanism of action, it was found that these compounds could interact with the main protease by forming hydrogen bonds, van der Waals forces, π-π stacking, etc., but compared with **N3**, the interacting groups were different. For example, the carbonyl, hydroxyl and amide bonds of the flavonoid fragment in preferred compound **11** could form multiple hydrogen bonds with HIS41, SER144, CYS145, and GLN192, respectively, but **N3** mainly formed hydrogen bonds with residues such as PHE140, HIS164, GLU166, GLN189, and THR190 of the main protease. However, residues such as HIS164, GLU166, GLN189, and THR190 could interact with compound **11** in van der Waals. Similarly, residues such as HIS41, SER144, and CYS145 could also interact with **N3** in other non-bonding ways. These results suggest that these compounds share the active pocket with **N3**, the mode of action and the group of action are different.

To further investigate the interaction stability of the 20 compounds screened by molecular docking with SARS-CoV-2 main protease, we used MD simulations to study the interaction of the top 20 compounds screened with protein receptors. The results showed that compounds **2**, **4**, **5**, **11**, **13**, **14**, **17**, and **18** had good binding effect on the target, and the binding free energy was less than −50 kJ/mol, which was worth further study. However, different interactions and different amino acid residues contribute differently to the total binding free energy. Especially for the preferred compound **11**, the van der Waals interaction and the electrostatic interaction are beneficial to the binding of the ligand to the receptor, and the van der Waals interaction is stronger than the electrostatic interaction. Polar solvation energies and non-polar solvation energies have opposite contributions to the binding free energy. Although non-polar solvation energies promote the binding of ligands to protein receptors, they account for a small proportion, while polar solvation energies are not conducive to the binding of ligands to receptors, but play a much greater role than non-polar solvation energies.

In addition, we also evaluated the drug-like properties of the 20 compounds obtained from the molecular docking screening according to the Lipinski rule. It was found that only compounds **8** and **9** followed these five rules, compounds **1**∼**4**, **10**∼**13**, **15**∼**17**, and **19** only followed 2 ~ 3 of these rules, while the others followed fewer rules. But that doesn’t mean that compounds that follow fewer rules can’t be drug candidates. For example, the anti-coronavirus drug Remdesivir only follows the two rules of the DruLiTo study, but plays an important role in fighting the virus. With the development of pharmaceutical technology, the use of new technologies can partially make up for the lack of certain properties of chemical molecules. Further, we also scored these 20 genistein derivatives for their biological activity against different targets. Among them, compounds **8**, **9** and compounds **11**, **12** could act on nuclear receptors and proteases, respectively, and the corresponding scores were all higher than 0 (meaning high activity), which indicates that key compounds **11** and **12** can be further developed as COVID-19 M^pro^ inhibitor. In addition to the activity analysis, we also analyzed ADMET for these 20 compounds, some of which also performed well. For example, in terms of gastrointestinal absorption, compounds **8** and **9** could achieve 100% gastrointestinal absorption. Metabolically, all compounds were incapable of acting on CYP2D6. Furthermore, except for compounds **4**, **8**, and **9**, the other compounds had no effect on CYP1A2 and CYP2C19. In terms of toxicity, none of the 20 compounds screened by molecular docking showed no effect on the skin. All compounds had no inhibitory effect on hERG1. None of the drug candidates exhibited AMES (a measure of a compound’s ability to induce DNA mutations) toxicity.

## Conclusion

In recent years, the COVID-19 has caused serious negative impact on life, health and economy around the world. At present, although the fatality rate of the SARS-CoV-2 has been significantly reduced, the persistent mutation of the virus has made it more infectious, which has adversely affected the effectiveness of vaccines (the main measures to deal with the epidemic at present) and poses a serious threat to the lives of elderly people with other chronic diseases. Therefore, the discovery of novel anti-COVID-19 drugs remains crucial. Soybeans are rich in biologically active substances, especially genistein compounds, which have therapeutic effects on many diseases. In this work, with COVID-19 M^pro^ as the target, two molecular docking software were applied to virtual screening of 9,614 small molecules, and the results were normalized to obtain 20 potential drug candidate molecules. Molecular dynamics simulation results showed that compounds **2**, **4**, **5**, **11**, **13**, **14**, **17**, and **18** had better binding free energy with the target. Further, the drug-likeness properties of the top 20 compounds were also analyzed, and found that plenty of compounds including **11** showed interesting drug-likeness properties. In addition, the inhibitory activity of these compounds was also studied using the Molinspiration Cheminformatics online server, and the results showed that compounds **11** and **12** had better protease inhibitory activity. Finally, the ADMET characteristics of the top 20 compounds were also predicted. All of the results show that compound **11** has the highest comprehensive advantage, which is very worthy of further research, and has high value for the future discovery and development of novel COVID-19 therapeutic drugs.

## Data Availability

The original contributions presented in the study are included in the article/Supplementary Material, further inquiries can be directed to the corresponding authors.
