# Supplementary material for: Discovery of genistein derivatives as potential SARS-CoV-2 main protease inhibitors by virtual screening, molecular dynamics simulations and ADMET analysis
Source: Front Pharmacol. 2022 Aug 25;13:961154. doi: 10.3389/fphar.2022.961154 (PMC9452787; doi:10.3389/fphar.2022.961154)
Supplement: Supplementary file 1 [file DataSheet1.docx]

Supplementary Information

Discovery of genistein derivatives as potential SARS-CoV‑2 main protease inhibitors by virtual screening, molecular dynamics simulations and ADMET analysis

Jiawei Liu^a^, Ling Zhang^b^, Jian Gao^c^, Baochen Zhang^a^, Xiaoli Liu^a^, Ninghui Yang^a^, Xiaotong Liu^a^, Xifu Liu^a,*^, Yu Cheng^a,*^

^a^*Center for Drug Innovation and Discovery, College of Life Science, Hebei Normal University, Shijiazhuang 050024, P.R. China*

^b^*School of Chemical Technology, Shijiazhuang University, Shijiazhuang 050035, P.R. China*

^c^*College of Plant Protection, Southwest University, Chongqing 400715, P.R. China*

* Corresponding author: Yu Cheng (Email: chengyu@hebtu.edu.cn); Xifu Liu (Email: xfliu@hebtu.edu.cn).

**1. 3D docked poses**


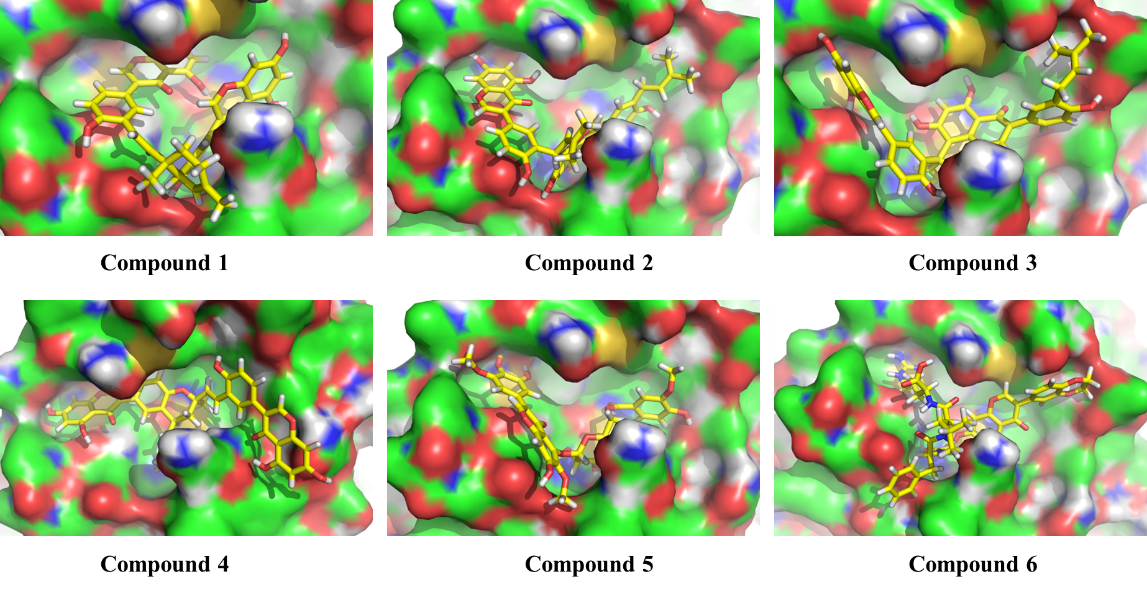


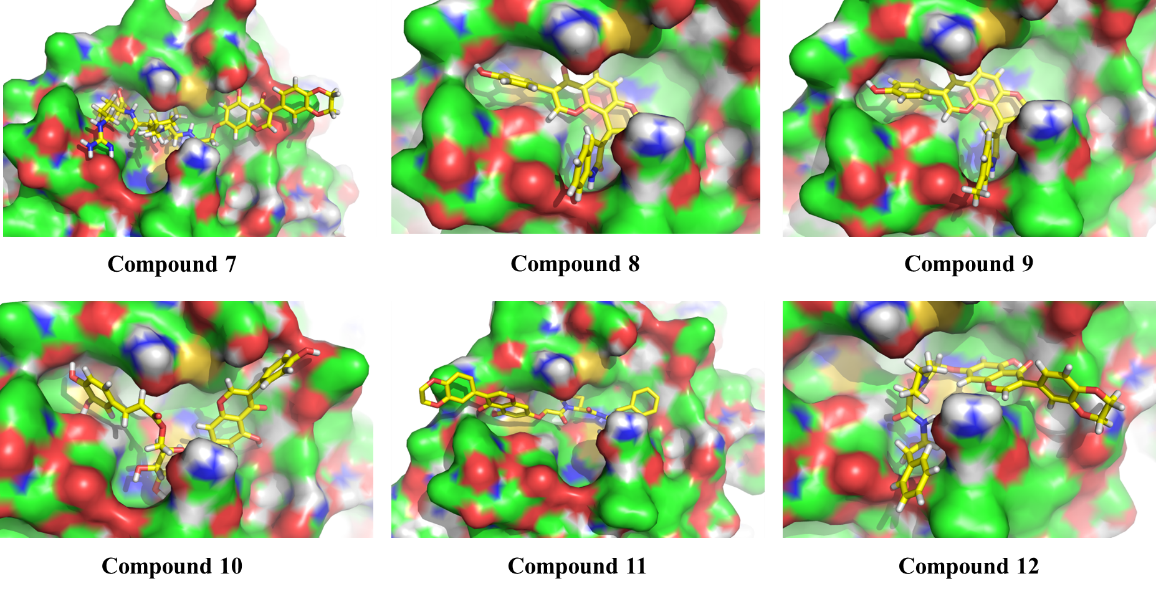


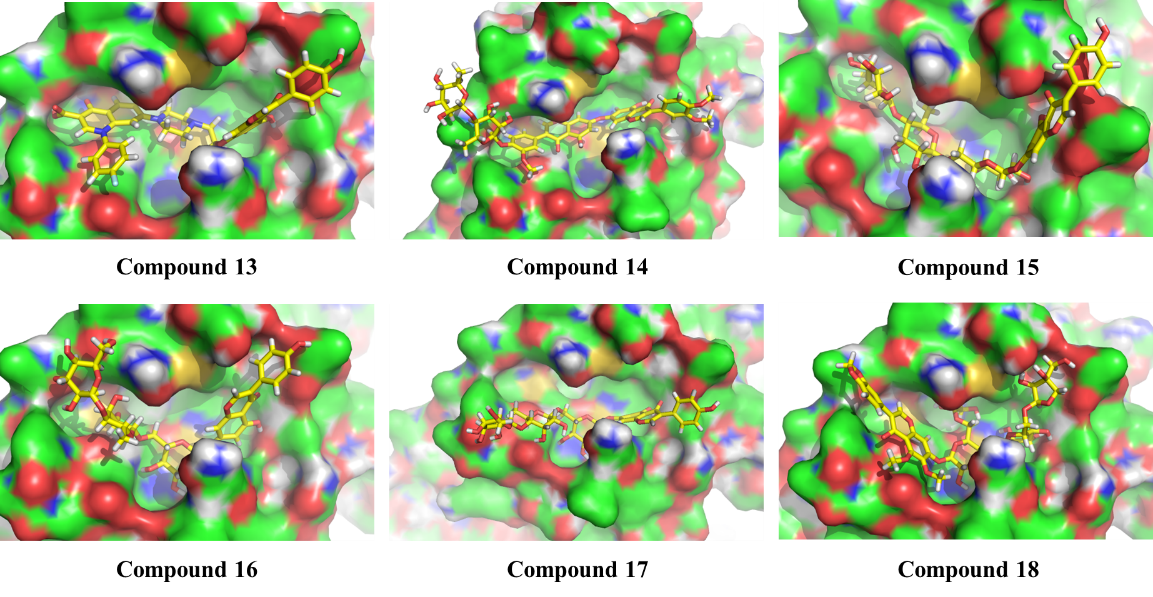


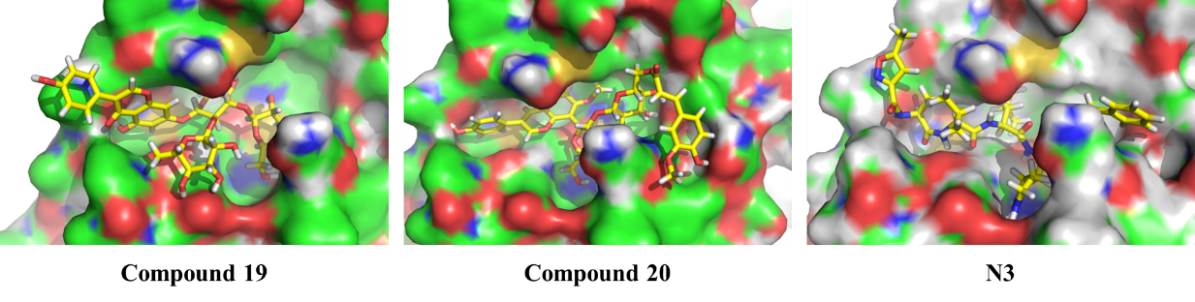


**Fig. S1.** Display of the final 3D docked poses for the top 20 genistein derivatives and **N3**.
